# 

**Published:** 1875-07

**Authors:** 


*The American Dental Convention.—*21*st Anniversary.*

The committee have made arrangements with the Proprietors of the *Ocean House, Long Branch*, for holding the *twenty-first anniversary* of the Convention at that place, on the 10th day of August, 1875, at 11 A. M.

The Messrs. Leland have, with their accustomed liberality offered us, (*free of charge*,) a large Hall suitable for our Meetings, also, to deduct 10 per cent. from the bills of members stopping at their House.

Ample arrangements have been made for the exhibition of instruments, implements, material, &c. All manufacturers and vendors are earnestly solicited to make the display as large and complete as possible.

The profession are cordially invited to be present at this, our Quarter Centennial Anniversary, believing it will be of unusual interest.

The Committee will secure rooms for those giving timely notice to the chairman.

J. G. AMBLER, *Chairman of Committee*,

25 *West* 23*d Street*, *N. Y.*

---

*American Dental Association.*

The Fifteenth Annual Meeting of the American Dental Association, will be held at Niagara Falls, New York, commencing on Tuesday, August 3d, 1875.

The Committee have up to this date completed the following arrangements:

The Cataract House will accommodate delegates at $4.00 per day, and the International at $3.50. Rooms will be reserved at either house for parties making known their desires a few days beforehand. Gentlemen accompanied by their wives should state this fact when ordering rooms.

The New York Central Rail Road will sell round-trip tickets from New York to the Falls and return for $17.50.

The Great Western Rail Road of Canada, will sell tickets for the round-trip at one full fare and a third. Parties desiring to avail themselves of these tickets, should ask for excursion tickets to American Dental Association.

Round-trip tickets from Chicago via. Michigan Central Rail Road, will be sold at $20.00, which is less than one and a third fare.

The Grand Trunk Rail Road will sell round-trip tickets at one fare and a third.

The C. C. C. & I. Rail Road will sell round-trip tickets at the rate of 4 cents a mile one way, to parties of 20 or more.

C. Stoddard Smith, *Rec. Secretary.*
G. L. Field, *Chairman Com. Arrangements.*

---

Boston, June 29th, 1875.

*To the Editor of the American Journal of Dental Science.*

Dear Sir.—At a Union Convention of the Massachusetts Dental Society and the Merrimack Valley Dental Association, the enclosed resolution was passed. In compliance with that note I forward it to you with the request to publish in your *Journal.*

Yours, truly,

Geo. F. Grant,
*Rec. Secretary Massachusetts Dental Society.*

---

*Resolved*, That we the members of the Massachusetts and Merrimack Valley Dental Societies, in Convention assembled, recommend to all dental practitioners the discontinuance of the use of Rubber as a base for artificial teeth, and the adoption in place thereof of Celluloid, believing this course would not only be for the best interests of ourselves but for our patients whom we serve.

*Voted.*—On motion of Dr. McDougal, that a copy of the above resolution be sent to all the dental journals of the country with a request to publish.

### TO READERS AND CORRESPONDENTS.

Communications intended for publication, and exchange journals and papers, should be addressed to the EDITOR OF THE AMERICAN JOURNAL OF DENTAL SCIENCE, 259 N. Eutaw St., Baltimore, Md. All letters relating to the business of the journal, or containing cash remittances, to be directed to Messrs. SNOWDEN & COWMAN. Articles for publication should be received by the editor at least three weeks previous to the first day of the month on which the number in which it is designed for them to appear, is issued.

### CONTENTS:

### ORIGINAL COMMUNICATIONS.

### MONTHLY SUMMARY.

The Diagnosis of the Lead-line .... 142
Cold Powder .... 142
Lead Poisoning in Lead Workers .... 143
Thymol an Antiseptic and Antifermentative Substance .... 143
Case of Abstention from Food .... 144

☞ The American Journal of Dental Science will contain fifty pages of reading matter in each number, making a volume of not less than

*Six Hundred* pages

EXCLUSIVE OF ADVERTISEMENTS.

THE

### AMERICAN JOURNAL

OF

### DENTAL SCIENCE.

**F. J. S. GORGAS, M. D., D. D. S., Editor.**

---

The *Journal* is issued on the first of each month and contains not less than *fifty* pages of reading matter in each number, exclusive of advertisements, making a volume of *six hundred* pages per annum.

In each number will be found Original Communications, Correspondence, Selected Articles, Monthly Summary, Bibliographical Notices and Editorial Matter, and every effort will be exerted to render the *Journal* worthy of the patronage of the Dental profession.

A generous co-operation is respectfully solicited from the members of the profession; and as the *Journal* has now a printing office of its own, which materially lessens the cost of its publication, it will be issued at the reduced subscription price of

**$2.50 Per Annum in Advance.**

Communications are respectfully solicited on all subjects pertaining to the practice of dentistry, as well as reports of cases occurring in dental practice.

---

### RATES OF ADVERTISING.

| | 1 MO. | 2 MO. | 3 MO. | 6 MO. | 12 MO. |
|---|---|---|---|---|---|
| 1 PAGE. | $15 00 | $22 50 | $30 00 | $50 00 | $100 00 |
| ½ " | 10 00 | 15 00 | 20 00 | 30 00 | 50 00 |
| ¼ " | 7 00 | 11 00 | 15 00 | 20 00 | 30 00 |
| ⅛ " | 5 00 | 8 00 | 11 00 | 15 00 | 20 00 |

All original communications designed for publication, works for review, new instruments for notice, exchanges, &c., should be directed to the editor, 259 N. Eutaw St., Baltimore, Md.

All advertisements and subscriptions should be addressed to

SNOWDEN & COWMAN,

*Publishers of the Am. Journal of Dental Science.*

86 W. FAYETTE ST. BET. CHARLES & LIBERTY STS.,

BALTIMORE, MD

